# Molecular Screening of Feline Glycogen Storage Disease Type II (Pompe Disease): Allele Frequencies of the *GAA*:c.1799G>A and c.55G>A Variants

**DOI:** 10.3390/genes16080938

**Published:** 2025-08-07

**Authors:** Abdullah Al Faruq, Tofazzal Md Rakib, Md Shafiqul Islam, Akira Yabuki, Shahnaj Pervin, Shinichiro Maki, Shigeki Tanaka, Nanami Arakawa, Osamu Yamato

**Affiliations:** 1 Laboratory of Clinical Pathology, Joint Faculty of Veterinary Medicine, Kagoshima University, Kagoshima 890-0065, Japan; faruqabdullahal103@gmail.com (A.A.F.); rakibtofazzal367@gmail.com (T.M.R.); si.mamun@ymail.com (M.S.I.); yabu@vet.kagoshima-u.ac.jp (A.Y.); s.pervin30@yahoo.com (S.P.); k6993382@kadai.jp (S.M.); k9829543@kadai.jp (N.A.); 2 Faculty of Veterinary Medicine, Chattogram Veterinary and Animal Sciences University, Chattogram 4225, Bangladesh; 3 Alpha Animal Hospital, Nagano 381-2226, Japan; alphaah@sea.plala.or.jp

**Keywords:** feline *GAA* gene, genotyping, Pompe disease, glycogen storage disease, variant

## Abstract

Background/Objectives: Glycogen storage disease type II, also known as Pompe disease (PD), is a rare autosomal recessive genetic disorder triggered by a deficiency in lysosomal acid α-glucosidase (GAA). Recently, we discovered two deleterious missense variants of the *GAA* gene, c.1799G>A (p.Arg600His) (a pathogenic mutation) and c.55G>A (p.Val19Met), in a domestic short-haired cat with PD. This study aimed to design genotyping assays for these two variants and ascertain their allele frequencies in Japanese cat populations. Methods: We developed fluorescent probe-based real-time polymerase chain reaction assays to genotype the c.1799G>A and c.55G>A variants. A total of 738 cats, comprising 99 purebred cats from 20 breeds and 540 mixed-breed cats, were screened using these assays. Results: Genotyping assays clearly differentiated all known genotypes of the two variants. None of the 738 cats tested carried the c.1799G>A variant. However, we identified cats with c.55G/A and c.55A/A genotypes in the purebred (A allele frequency: 0.081) and mixed-breed cats (0.473). A significant difference (*p* < 0.001) was observed in the A allele frequency between the two groups. Conclusions: The c.1799G>A mutation appears rare in cat populations, suggesting it may be confined to specific pedigree Japanese mixed-breed cats. The c.55G>A variant was detected in purebred and mixed-breed cats, suggesting that it may not be directly linked to feline PD. However, additional studies are required to elucidate the precise relationship between this variant and cardiac function. Genotyping assays will serve as valuable tools for diagnosing and genotyping feline PD.

## 1. Introduction

Glycogen storage disease type II (MIM #232300), commonly known as Pompe disease (PD), is an autosomal recessive progressive neuromuscular disorder induced by a deficiency in lysosomal acid α-glucosidase (GAA, EC 3.2.1.20) [1,2,3,4,5,6]. This enzyme, encoded by the *GAA* gene, degrades α-1,4 and α-1,6 linkages of glycogen, maltose, and isomaltose within lysosomes. A deficiency in this glycogen-degrading enzyme results in the gradual accumulation of cytoplasmic glycogen-filled lysosomes in various tissues. The most severely affected tissues include the cardiac and skeletal muscles, cerebral cortex, and esophageal smooth muscle. Clinical manifestations of PD encompass acute tachypnea, poor growth, hypothermia, lethargy, and cardiomegaly, accompanied by mild pleural effusion, atrial dilatation, and left ventricular systolic dysfunction.

In humans, PD has been broadly categorized into two forms based on age of onset and severity [7,8,9,10]. Infantile-onset PD (IOPD) is the more severe form, presenting symptoms within the first few weeks to months of infancy. However, it is characterized by generalized muscle weakness, respiratory distress, hypertrophic cardiomyopathy, hypotonia, macroglossia, and a high mortality rate due to cardiorespiratory failure, typically before two years of age. Generalized muscular glycogenosis, cardiac hypertrophy, and hepatomegaly have been documented as pathological features of IOPD [11,12]. Late-onset PD (LOPD) is more common than IOPD and typically manifests after 12 months of age or as late as the sixth decade of life, and it is characterized by a gradual weakening of primarily the proximal limb and respiratory muscles, leading to significant morbidity and mortality [3,4,5]. Associated conditions such as elevated serum creatine kinase activity, chronic respiratory failure, atrial aneurysm, oropharyngeal dysphagia, scoliosis, and ptosis have been reported in cases of human LOPD [5,12,13].

PD has been reported in various animals, including cats (OMIA 000419-9685) [14,15,16,17,18,19,20,21,22,23,24,25,26,27]. Pathogenic mutations have been identified in cattle [20,21], dogs [15], and cats [24]. The first feline mutation was recently identified in a domestic short-haired (DSH; mixed-breed) cat diagnosed with PD [14]. The homozygous causative mutation, *GAA*:c.1799G>A, results in an amino acid substitution (p.Arg600His) in the feline GAA protein [24]. The codon position aligns with three human missense mutations (p.Arg600Cys, p.Arg600Leu, and p.Arg600His) associated with human IOPD. A preliminary survey was conducted on 100 clinically healthy mixed-breed cats using a polymerase chain reaction (PCR)-restriction fragment length polymorphism (RFLP) assay and showed no presence of the c.1799G>A variant. This finding suggests that the c.1799G>A mutation is pathogenic in feline PD. However, the exact frequency of this mutant allele has not yet been determined in large-scale populations of both purebred and mixed-breed cats.

Moreover, during our analysis of the *GAA* gene in this PD-affected cat, we identified an additional homozygous missense variant (c.55G>A), resulting in p.Val19Met [24]. No human mutation has yet been found at this amino acid position. The variant was predicted to be deleterious by several pathogenicity and stability predictors and was associated with a moderately low sorting intolerant from tolerant (SIFT) score (0.03). However, the correlation of the c.55G>A variant with feline PD and its allele frequency in the cat population remains to be established.

This study aimed to establish rapid genotyping assays for these two variants and conduct a large-scale survey involving purebred and mixed-breed cats. In addition, the aim was to elucidate the allele frequencies and their correlation with feline PD.

## 2. Materials and Methods

### 2.1. Samples

A total of 738 whole-blood specimens from 99 purebred, 540 mixed, and 99 unknown-breed cats were analyzed in this study. The purebred cats represented 20 different breeds. Our laboratory stored all blood specimens on Flinders Technology Associates (FTA) cards (QIAcard FTA Classic; Qiagen, Hilden, Germany). These were used as control samples from clinically healthy cats or for diagnosing various suspected genetic disorders. The experiments conducted in this study adhered to the Guidelines Regulating Animal Use and Ethics at Kagoshima University (No. VM15041; approval date: 29 September 2015).

### 2.2. Preparation of the DNA Template

A disk with a diameter of 1.2 mm was punched from the FTA cards containing blood spots using a hole puncher. The disk was then placed into a separate 0.2 mL tube, and DNA was extracted using a commercial kit (DNA Extract All Reagents Kit; Applied Biosystems, Foster City, CA, USA). The punch was lysed within the tube using 8 µL of lysis solution and subsequently incubated at 95 °C for 3 min. Following this, 8 µL of stabilizing solution from the kit was added to the tube. The DNA-containing solution (DNA template) was then transferred to a new 0.2 mL tube and stored at −25 °C until further analysis.

### 2.3. Real-Time PCR Genotyping

Amplifications were performed using a real-time PCR system (StepOne; Applied Biosystems). Two sets of specific primer pairs and TaqMan probes with minor groove binder (MGB) (Applied Biosystems), linked to each fluorescent reporter dye (6-carboxyrhodamine or 6-carboxyfluorescein) at the 5′-end and a non-fluorescent quencher dye at the 3′-end, were used (Table 1). The amplifications were performed in a final volume of 10 µL. This volume comprised a master mix (2×) (GTXpress Master Mix; Applied Biosystems), a genotyping assay mix (80×) including specific primers and TaqMan MGB probes (Table 1), nuclease-free water, and 2 µL of the DNA template. The cycling conditions were set as follows: 20 s at 95 °C, followed by 50 cycles, each consisting of 3 s at 95 °C and 20 s at 60 °C. The holding stage post-PCR was set at 25 °C for 30 s. For the validation of these real-time PCR assays, we utilized control DNA samples (c.1799G/G, c.1799A/A [PD-affected cat, as reported previously], c.55G/G, c.55G/A, and c.55A/A genotypes), which were previously confirmed by Sanger sequencing and PCR-RFLP assay in our earlier study [24].

### 2.4. Statistical Analysis

Statistical analyses were performed using R (version 4.5.0). Differences in allele frequencies between purebred and mixed-breed cats were statistically analyzed using Fisher’s exact test. *p* values less than 0.05 were considered to indicate a statistically significant difference.

## 3. Results

**Figure 1:**
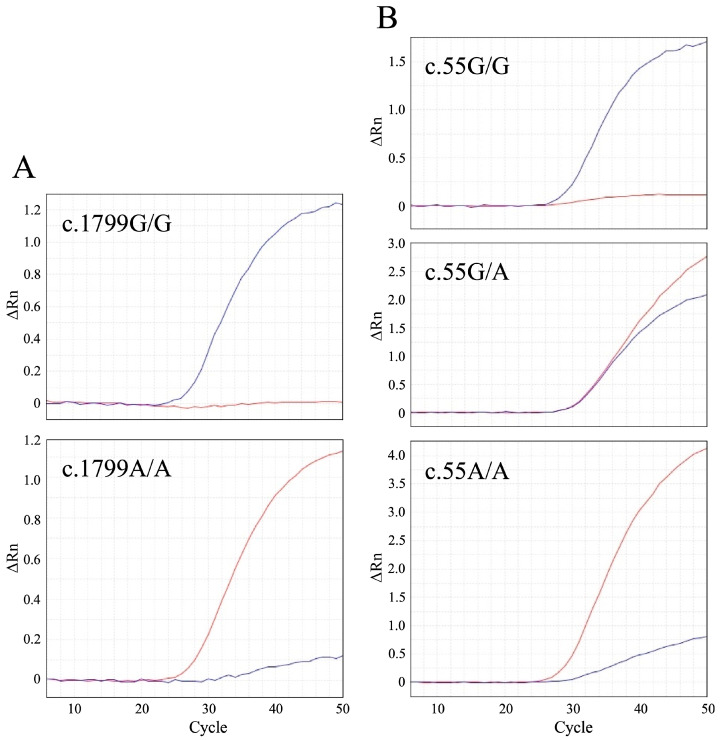
Real-time polymerase chain reaction (PCR) amplification plots of the *GAA*:c.1799G>A (**A**) and c.55G>A variants (**B**) associated with feline Pompe disease. Amplification is graphed as fluorescence intensity (ΔRn values) versus cycle number. ΔRn values denote the reporter dye signal, normalized to the internal reference dye, and adjusted for the baseline signal determined in the initial cycles of PCR. The two amplification plots for c.1799G>A depict the c.1799G/G and A/A genotypes, while the three plots for c.55G>A represent the c.55G/G, G/A, and A/A genotypes. The blue and red lines signify amplification in the presence of wild-type and mutant alleles, respectively.

We developed TaqMan MGB probe-based real-time PCR assays (Table 1). We validated these methods using control samples, including c.1799G/G, c.1799A/A, c.55G/G, c.55G/A, and c.55A/A genotypes (Figure 1). The results demonstrated that these real-time PCR assays effectively differentiated all known genotypes, except for c.1799G/A due to its unavailability.

The findings from our survey of cat populations are summarized in Table 2. None of the 738 examined cats, comprising 99 purebred cats from 20 breeds and 540 mixed-breed cats, carried the c.1799G>A variant, a pathogenic mutation in feline PD. In contrast, for the c.55G>A variant, 86 out of 99 purebred cats (86.9%) were G/G, 10 (10.1%) were G/A, and 3 (3.0%) were A/A. Cats carrying the c.55G>A variant were observed in 7 of the 20 breeds. In the mixed-breed population, 189 cats (35.0%) were G/G, 191 (35.4%) were G/A, and 160 (29.6%) were A/A. The frequency of the A allele for the c.55G>A variant was 0.081 in purebred cats and 0.473 in mixed-breed cats. Additionally, a statistically significant difference (*p* < 0.001) in these allele frequencies was identified using Fisher’s exact test.

## 4. Discussion

Feline PD was first documented in a cat in 1969 [27]. However, molecular characterization was not conducted at that time. In 2022, our research group identified an eight-month-old DSH cat with severe heart failure, marking another feline case with PD [14]. Our subject’s diagnosis was primarily based on clinical and pathological features analogous to those observed in human patients with PD. Our subject first showed symptoms at eight months of age, presenting with poor growth, acute tachypnea, hypothermia, lethargy, and recumbency at an animal hospital after a three-month history of unexplained fever. A radiographic examination revealed cardiomegaly in the cat. Furthermore, echocardiography showed dilation of both the atria and left ventricular systolic dysfunction. The cat eventually succumbed to pulmonary edema caused by chronic heart failure. Postmortem histopathological examination disclosed severe vacuolation of the cardiac muscle cells, filled with coarse glycogen granules. These severe clinical and pathological manifestations in the cat resembled those of human IOPD. In addition, we recently detected a homozygous pathogenic mutation (*GAA*:c.1799G>A) in this cat with PD, leading to a deleterious amino acid substitution (p.Arg600His), which is observed in human IOPD [24]. The severity of clinical and pathological manifestations mirrored the deleterious characteristics of the mutation, as evaluated by various pathogenicity and stability predictors, including an extremely low SIFT score (0.00).

In this study, none of the 738 cats tested, including 99 purebred and 540 mixed-breed cats, carried the c.1799G>A mutation. The observation suggests that mutations responsible for severe feline PD are rare in the general cat population. The mutation may be confined to specific pedigrees of Japanese mixed-breed cats. Only one DSH cat with sporadic PD has been identified in a localized area of Japan. We previously discovered a DSH cat with sporadic dihydropyrimidinase deficiency (OMIA 001776-9685), homozygous for a missense mutation (*DPYS*:c.1303G>A) [28]. A population screening of 1000 mixed-breed cats did not detect any cats with the *DPYS*:c.1303G>A mutation. Furthermore, we identified several DSH cats with GM2 gangliosidosis variant 0 (Sandhoff disease, OMIA 001462-9685), all of which were homozygous for a nonsense mutation (*HEXB*:c.667C>T) [29]. Another screening of 1015 mixed-breed cats revealed no individuals with the *HEXB*:c.667C>T mutation [29]. However, a survey of erythrocyte pyruvate kinase deficiency (OMIA 000844-9685) revealed mutant allele frequencies of the *PKLR*:c.707-53G>A mutation to be 0.214 in Abyssinian cats, 0.229 in Somali cats, 0.016 in Bengal cats, and 0.008 in American Shorthair cats [30]. The difference in mutant allele frequency between purebred and mixed-breed cats may be attributed to the variations in the genetic diversity of the two populations.

The screening was additionally performed for another deleterious missense variant (*GAA*:c.55G>A). This was undertaken because an amino acid substitution (p.Val19Met) induced by this variant was previously assessed as deleterious or unstable by seven out of the eleven predictors used [24]. Consequently, this variant was hypothesized to be linked to a milder form of feline PD similar to human LOPD. Screening results demonstrated a relatively high frequency of this variant, 0.081 and 0.473, in purebred and mixed-breed cats, respectively (Table 2). The significant difference in frequency between the two populations might be due to stratification caused by selective breeding in purebred cats. Approximately 30% of mixed-breed cats were homozygous for this variant. To our knowledge, most cats do not exhibit any diseases or disorders, including cardiac dysfunction. These findings suggest that the c.55G>A variant may not be directly associated with feline PD. However, this study did not investigate the cardiac function of cats homozygous for this variant.

We devised two sets of real-time PCR assays (Table 1) to identify the c.1799G>A and c.55G>A variants, which can distinctly differentiate all known genotypes linked with these two variants (Figure 1). These genotyping assays are valuable tools for diagnosing and genotyping feline PD and related disorders.

## 5. Conclusions

In conclusion, the c.1799G>A mutation, a pathogenic mutation associated with feline PD, is rarely observed in the cat populations, indicating that it may be confined to specific pedigree Japanese mixed-breed cats. The c.55G>A variant, another missense variant that is predicted to be deleterious, is observed at a relatively high frequency in purebred and mixed-breed cats. This observation suggests it may not directly correlate with feline PD or evident cardiac dysfunction. Additional research is necessary to determine the precise relationship between this variant and feline cardiac function. Further studies are required to evaluate the function of the c.55G>A variant by measuring the GAA activity of tissues from cats homozygous for this variant.

## Figures and Tables

**Table 1:** Characteristics and sequencing of primers and probes used in this study.

Variant	Primer/Probe	Sequence (5′ to 3′)	Reporter (5′)	Quencher (3′)	Conc. (nM)
c.1799G>A	Forward primer	CCAGGGCCCTGGTCAAG	NA	NA	450
	Reverse primer	AGTGGCCGGCGTATCG	NA	NA	450
	Wild-type probe	TGATCTCCC**G**CTCGACC	VIC	NFQ	100
	Mutant-type probe	TGATCTCCC**A**CTCGACC	FAM	NFQ	100
c.55G>A	Forward primer	GTGGCCGCTGTGCTG	NA	NA	450
	Reverse primer	GGACGTGCCCCAGGAG	NA	NA	450
	Wild-type probe	CTACATCCTC**G**TGTCCC	VIC	NFQ	100
	Mutant-type probe	ACATCCTC**A**TGTCCC	FAM	NFQ	100

The positions of the variants are indicated in boldface. NA, not applicable; FAM, 6-carboxyfluorescein; VIC, 6-carboxyrhodamine; NFQ, non-fluorescent quencher; Conc., concentration.

**Table 2:** Screening of the *GAA*:c.55G>A variant in the populations of purebred and mixed-breed cats.

Breed	Number Examined	c.55G/G	c.55G/A	c.55A/A	Frequency of A Allele
Abyssinian	10	10	0	0	0
American Curl	3	3	0	0	0
American Shorthair	16	12	3	1	0.156
Bengal	3	2	1	0	0.117
Chartreux	1	1	0	0	0
Chinchilla	16	16	0	0	0
Devon Rex	1	1	0	0	0
Exotic	2	0	0	0	0
Himalayan	1	1	0	0	0
La Perm	4	3	1	0	0.125
Maine Coon	5	2	3	0	0.3
Munchkin	2	1	1	0	0.25
Norwegian Forest Cat	3	0	0	0	0
Persian	4	0	0	0	0
Ragamuffin	1	0	0	0	0
Ragdoll	2	0	0	0	0
Russian Blue	3	0	0	0	0
Scottish Fold	12	11	1	0	0.042
Somali	8	8	0	0	0
Tonkinese	2	0	0	2	1
Purebred (Total)	99	86	10	3	0.081 *
Mixed-breed	540	189	191	160	0.473 *
Unknown	99	61	26	12	0.253
Total	738	336	227	175	0.391

* Differences in allele frequencies between purebred and mixed-breed cats were statistically significant (*p* < 0.001) using Fisher’s exact test.

## Data Availability

The original contributions presented in this study are included in the article. Further inquiries can be directed to the corresponding author.

## Abbreviations

The following abbreviations are used in this manuscript:

PD	Pompe disease
IOPD	infantile-onset Pompe disease
LOPD	late-onset Pompe disease
PCR	polymerase chain reaction
RFLP	restriction fragment length polymorphism
SIFT	sorting intolerant from tolerant
FTA	Flinders Technology Associates
MGB	minor groove binder

## References

[1] van der Ploeg A.T., Reuser A.J.J. (2008). Pompe’s disease. Lancet.

[2] Almodóvar-Payá A., Villarreal-Salazar M., de Luna N., Nogales-Gadea G., Alberto Real-Martínez A., Andreu A.L., Miguel Angel Martín M.A., Arenas J., Lucia A., Vissing J. (2020). Preclinical research in glycogen storage diseases: A comprehensive review of current animal models. Int. J. Mol. Sci..

[3] Meena N.K., Raben N. (2020). Pompe disease: New developments in an old lysosomal storage disorder. Biomolecules.

[4] Kishnani P.S., Amartino H.M., Lindberg C., Miller T.M., Wilson A., Keutzer J. (2014). Methods of diagnosis of patients with Pompe disease: Data from the Pompe registry. Mol. Genet. Metab..

[5] Almeida V., Conceição I., Fineza I., Coelho T., Silveira F., Santos M., Valverde A., Geraldo A., Maré R., Aguiar T.C. (2017). Screening for Pompe disease in a Portuguese high risk population. Neuromuscul. Disord..

[6] Pillai N.R., Fabie N.A.V., Kaye T.V., Rosendahl S.D., Ahmed A., Hietala A.D., Jorgenson A.B., Lanpher B.C., Whitley C.B. (2023). Disparities in late and lost: Pediatricians’ role in following Pompe disease identified by newborn screening. Mol. Genet. Metab..

[7] Fukuhara Y., Fuji N., Yamazaki N., Hirakiyama A., Kamioka T., Seo J.H., Mashima R., Kosuga M., Okuyama T. (2018). A molecular analysis of the *GAA* gene and clinical spectrum in 38 patients with Pompe disease in Japan. Mol. Genet. Metab. Rep..

[8] Thirumal Kumar D., Umer Niazullah M., Tasneem S., Judith E., Susmita B., George Priya Doss C., Selvarajan E., Zayed H. (2019). A computational method to characterize the missense mutations in the catalytic domain of GAA protein causing Pompe disease. J. Cell. Biochem..

[9] Kishnani P.S., Beckemeyer A.A., Mendelsohn N.J. (2012). The new era of Pompe disease: Advances in the detection, understanding of the phenotypic spectrum, pathophysiology, and management. Am. J. Med. Genet. C Semin. Med. Genet..

[10] Chan J., Desai A.K., Kazi Z.B., Corey K., Austin S., Hobson-Webb L.D., Case L.E., Jones H.N., Kishnani P.S. (2017). The emerging phenotype of late-onset Pompe disease: A systematic literature review. Mol. Genet. Metab..

[11] Smith L.D., Bainbridge M.N., Parad R.B., Bhattacharjee A. (2020). Second tier molecular genetic testing in newborn screening for Pompe disease: Landscape and challenges. Int. J. Neonatal. Screen..

[12] Dasouki M., Jawdat O., Almadhoun O., Pasnoor M., McVey A.L., Abuzinadah A., Herbelin L., Barohn R.J., Dimachkie M.M. (2014). Pompe disease: Literature review and case series. Neurol. Clin..

[13] Ravaglia S., De Filippi P., Cirio S., Danesino C., Moggio M., Mongini T., Maggi L., Servidei S., Vianello A., Toscano A. (2021). Polymorphism in exercise genes and respiratory function in late-onset Pompe disease. J. Appl. Physiol..

[14] Tanaka S., Suzuki R., Koyama H., Machida N., Yabuki A., Yamato O. (2022). Glycogen storage disease in a young cat with heart failure. J. Vet. Intern. Med..

[15] Seppälä E.H., Reuser A.J., Lohi H. (2013). A nonsense mutation in the acid α-glucosidase gene causes Pompe disease in Finnish and Swedish Lapphunds. PLoS ONE.

[16] Manktelow B., Hartley W. (1975). Generalized glycogen storage disease in sheep. J. Comp. Pathol..

[17] Jolly R., Van-de-Water N., Richards R., Dorling P.R. (1977). Generalized glycogenosis in beef shorthorn cattle—Heterozygote detection. Aust. J. Exp. Biol. Med. Sci..

[18] O’sullivan B., Healy P., Fraser I., Nieper R.E., Whittle R.J., Sewell C.A. (1981). Generalised glycogenosis in Brahman cattle. Aust. Vet. J..

[19] Matsui T., Kuroda S., Mizutani M., Kiuchi K., Suzuki K., Ono T. (1983). Generalized glycogen storage disease in Japanese quail (*Coturnix coturnix japonica*). Vet. Pathol..

[20] Čítek J., ŘEhout V., Večerek L., Hájková J. (2007). Genotyping glycogen storage disease type II and type V in cattle reared in the Czech Republic. J. Vet. Med. A Physiol. Pathol. Clin. Med..

[21] Dennis J.A., Moran C., Healy P.J. (2000). The bovine α-glucosidase gene: Coding region, genomic structure, and mutations that cause bovine generalized glycogenosis. Mamm. Genome.

[22] Lyons R.E., Johnston D.J., McGowan M.R., Laing A.R., Robinson B., Owen H., Hill B.D., Burns B.M. (2017). E7 (1057ΔTA) mutation of the acidic α-glucosidase gene causes Pompe’s disease in Droughtmaster cattle. Aust. Vet. J..

[23] Dennis J.A., Healy P.J., Reichmann K.G. (2002). Genotyping Brahman cattle for generalised glycogenosis. Aust. Vet. J..

[24] Rakib T.M., Islam M.S., Tanaka S., Yabuki A., Pervin S., Maki S., Faruq A.A., Tacharina M.R., Yamato O. (2023). Novel mutation in the feline *GAA* gene in a cat with glycogen storage disease type II (Pompe disease). Animals.

[25] Walvoort H.C., Slee R.G., Koster J.F. (1982). Canine glycogen storage disease type II: A biochemical study of an acid α-glucosidase-deficient Lapland dog. Biochim. Biophys. Acta.

[26] Skelly B.J., Franklin R.J.M. (2002). Recognition and diagnosis of lysosomal storage diseases in the cat and dog. J. Vet. Intern. Med..

[27] Sandström B., Westman J., Öckerman P. (1969). Glycogenosis of the central nervous system in the cat. Acta. Neuropathol..

[28] Chang H.S., Shibata T., Arai S., Zhang C., Yabuki A., Mitani S., Higo T., Sunagawa K., Mizukami K., Yamato O. (2012). Dihydropyrimidinase deficiency: The first feline case of dihydropyrimidinuria with clinical and molecular findings. JIMD Rep..

[29] Rahman M.M., Shoubudani T., Mizukami K., Chang H.S., Hossain M.A., Yabuki A., Mitani S., Higo T., Arai T., Yamato O. (2011). Rapid and simple polymerase chain reaction-based diagnostic assays for GM2 gangliosidosis variant 0 (Sandhoff-like disease) in Japanese domestic cats. J. Vet. Diagn. Investig..

[30] Kushida K., Giger U., Tsutsui T., Inaba M., Konno Y., Hayashi K., Noguchi K., Yabuki A., Mizukami K., Kohyama M. (2015). Real-time PCR genotyping assay for feline erythrocyte pyruvate kinase deficiency and mutant allele frequency in purebred cats in Japan. J. Vet. Med. Sci..

